# Hemispheric differences between left and right supramarginal gyrus for pitch and rhythm memory

**DOI:** 10.1038/srep42456

**Published:** 2017-02-15

**Authors:** Nora K. Schaal, Bettina Pollok, Michael J. Banissy

**Affiliations:** 1 Department of Experimental Psychology, Heinrich-Heine-University, 40225 Düsseldorf, Germany; 2 Institute of Clinical Neuroscience and Medical Psychology, Medical Faculty, Heinrich-Heine-University, 40225 Düsseldorf, Germany; 3 Department of Psychology, Goldsmiths, University of London, SE14 6NW, London, UK

## Abstract

Functional brain imaging studies and non-invasive brain stimulation methods have shown the importance of the left supramarginal gyrus (SMG) for pitch memory. The extent to which this brain region plays a crucial role in memory for other auditory material remains unclear. Here, we sought to investigate the role of the left and right SMG in pitch and rhythm memory in non-musicians. Anodal or sham transcranial direct current stimulation (tDCS) was applied over the left SMG (Experiment 1) and right SMG (Experiment 2) in two different sessions. In each session participants completed a pitch and rhythm recognition memory task immediately after tDCS. A significant facilitation of pitch memory was revealed when anodal stimulation was applied over the left SMG. No significant effects on pitch memory were found for anodal tDCS over the right SMG or sham condition. For rhythm memory the opposite pattern was found; anodal tDCS over the right SMG led to an improvement in performance, but anodal tDCS over the left SMG had no significant effect. These results highlight a different hemispheric involvement of the SMG in auditory memory processing depending on auditory material that is encoded.

Pitch and rhythm are two important factors for music perception and cognition^1^. They are related to language production and comprehension^2^,^3^,^4^, and are associated with auditory verbal and non-verbal memory^1^,^5^,^6^. With this in mind, factors contributing to pitch and rhythm memory have received considerable interest, and functional brain imaging studies have highlighted underlying neural structures for pitch and rhythm memory. For pitch memory a complex neural network has been revealed including frontal, temporal and parietal areas^7^,^8^,^9^,^10^. Of particular relevance to the current study is the supramarginal gyrus (SMG), which has been shown to relate to inter-individual variation in pitch memory performance^11^. Rhythm memory has been associated with a similar frontal-parietal network (including brain activation in the supramarginal gyrus) as pitch memory^9^,^12^,^13^, as well as activity in the cerebellum and supplementary motor area^12^,^13^, anterior insular cortex and left anterior cingulate gyrus^9^.

Investigations of pitch and rhythm memory in the same study are sparse, and thus overlapping and separable brain areas related to rhythm and pitch memory are fairly unknown. Jerde and colleagues^9^ explored the neural correlates of pitch and rhythm working memory in non-musicians and revealed distinct neural circuits for each process. Whereas pitch memory showed activation in a right hemisphere network of frontal, parietal and temporal areas, rhythm memory was associated with activation in the cerebellar hemispheres and vermis, right anterior insular cortex, and left anterior cingulate gyrus. Additionally, and importantly in the context of the present study, the SMG has been shown to have differential laterisation of function according to whether rhythm or pitch word devision wrong tasks were completed. Jerde and colleagues revealed activation in the inferior parietal lobe (the area of the SMG) bilaterally for pitch memory and only right hemisphere activation for rhythm memory^9^.

Further evidence for a distinction of rhythm and pitch processing can be gained from studies investigating impairments in each process. For instance, in congenital amusia (individuals with deficits in music processing and memory) there are a number of cases where individuals show selective impairment in pitch processing, but not rhythm processing^14^,^15^. Additionally, brain-lesion studies exploring rhythm and pitch discrimination have shown that rhythm perception can be disturbed, whereas pitch perception is intact and vice versa^16^,^17^,^18^,^19^. Taken together, these studies indicate that pitch and rhythm processing can be dissociated, and therefore neural processes related to encoding pitch and rhythm may rely on distinct neural circuits.

With regards to the neural network for pitch and rhythm memory, brain imaging alone cannot tell us about causal relationships between brain areas and behaviour. For this alternative methods that permit the investigation of modulations in brain activity are more powerful (e.g. lesions^20^,^21^,^22^; brain stimulation^23^,^24^,^25^,^26^,^27^,^28^). In this context, transcranial direct current stimulation (tDCS) has been shown to be a promising tool to investigate the causal role of specific brain areas for cognitive tasks^29^,^30^. TDCS uses two stimulation modes, anodal and cathodal stimulation. In most studies, anodal tDCS enhances cortical excitability in the targeted area, while cathodal tDCS typically suppresses cortical excitability^31^,^32^,^33^,^34^. However, also some contrary effects depending on the duration and intensity of the stimulation input have been shown more recently^35^ and there is an ongoing discussion about the reliability and efficiency of tDCS protocols depending on a number of trait and state variables^36^,^37^. With regards to pitch memory, studies using non-invasive brain stimulation methods have consistently revealed a critical role for the left SMG for pitch memory in non-musicians^38^,^39^,^40^,^41^. To date, no brain stimulation studies have been conducted to examine the neural mechanisms of rhythm memory. Therefore we sought to investigate this for the first time in the present study.

More specifically, the aim of this study was to examine the role of the left and right SMG for rhythm and pitch memory. As noted above, prior brain stimulation work has indicated a causal role for the left SMG in pitch memory^38^,^41^, but whether this region plays a similar role in rhythm memory remains unclear. Previous functional magnetic resonance imaging (fMRI) findings on the role of the SMG in rhythm memory paint a mixed picture: in one study bilateral activation of the SMG was found^13^, in others activation of the right SMG has been reported^9^,^12^,^42^, and another study highlighted left hemisphere activation of the SMG^43^. We sought to examine the influence of tDCS targeted at left and right SMG on rythym and pitch memory to disentangle the hemispheric specialisation of the SMG for rhythm and pitch memory. Two experiments were conducted. In Experiment 1 we investigated whether the left SMG is specifically involved in pitch memory or whether it is also significant in another auditory memory domain, such as rhythm memory. In Experiment 2, we explored whether the right SMG could be linked to rhythm memory. Participants took part in two sessions and either anodal or sham tDCS over the left SMG (Experiment 1) and right SMG (Experiment 2) was applied. After stimulation, participants completed a pitch and rhythm span task. Based on previous research^38^,^40^, an improvement of pitch memory after anodal tDCS over the left SMG was expected. Regarding rhythm memory, an effect of anodal tDCS on memory performance was hypothesised as brain imaging studies show the involvement of the SMG for rhythm memory^12^,^13^,^43^, but the lateralisation of the effect is less predictable.

## Results

An overall mixed-factor ANOVA with the within-subject variables *stimulation* (sham vs. anodal) and *task* (pitch span vs. rhythm span) and the between-subject variable *group* (left SMG group vs. right SMG group) was conducted. The analysis revealed a significant main effect for *task* [*F*(1,40) = 167.98, *p* < 0.001, 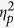 = 0.808] and non-significant main effects for *stimulation* [*F*(1,40) = 1.77, *p* = 0.191, 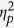 = 0.042] and *group* [*F*(1,28) = 2.82, *p* = 0.104, 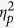 = 0.091]. A trend for the *stimulation* * *group* interaction was found [*F*(1,40) = 3.61, *p* = 0.065, 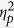 = 0.083] and there were non-significant results for the *task* * *group* interaction [*F*(1,40) = 1.14, *p* = 0.291, 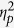 = 0.028] and *stimulation* * *task* interaction [*F*(1,40) = 0.059, *p* = 0.809, 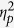 = 0.001]. Importantly, the three-way *stimulation* * *task* * *group* interaction was significant [*F*(1,40) = 11.33, *p* = 0.002, 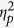 = 0.221].

**Figure 1:**
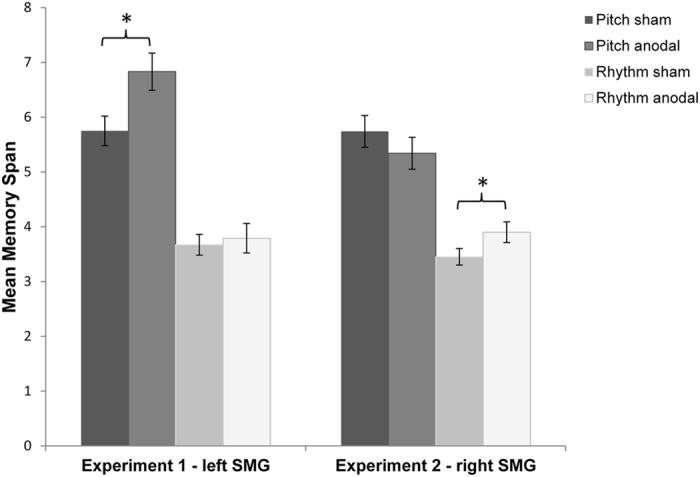
Overview of results. In Experiment 1 anodal tDCS over the *left* SMG led to a facilitation of pitch memory, whereas rhythm memory was not affected. However, anodal tDCS over the *right* SMG improved rhythm memory in Experiment 2, whereas pitch memory was not significantly modulated by the stimulation. The error bars represent SEM.

In order to disentangle this interaction, two ANOVAs were calculated separately for each group with the within-subject variables *stimulation* and *task*. In the group receiving stimulation over the left SMG (Experiment 1) significant main effects of *stimulation* [*F*(1,19) = 4.80, *p* = 0.041, 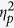 = 0.202] and *task* [*F*(1,19) = 106.46, *p* < 0.001, 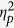 = 0.849] were revealed, indicating that overall participants were slightly better on both tasks in the anodal condition compared to sham condition, and that participants were better at the pitch memory task compared to rhythm memory performance. Additionally, the *stimulation* * *task* interaction was also significant [*F*(1,19) = 4.52, *p* = 0.047, 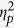 = 0.192]. To examine this further, planned paired samples t-tests with Bonferroni correction comparing performances after sham and anodal stimulation on the pitch memory and rhythm memory task were conducted. The comparison of pitch memory performance between sham and anodal tDCS revealed a significant difference [*t*(19) = 2.61, *p* = 0.017, 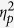 = 0.264] (Fig. 1). The participants performed significantly better on the pitch memory span task after receiving anodal tDCS over the left SMG compared to sham stimulation. No difference was found for the rhythm task [*t*(19) = 0.064, *p* = 0.950, 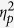 = 0.021].

For Experiment 2 (stimulation over the right SMG) a non-significant main effect of *stimulation* [*F*(1,21) = 0.18, *p* = 0.678, 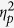 = 0.008] and a significant main effect of *task* [*F*(1,21) = 67.10, *p* < 0.001, 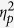 = 0.762] were found. The *stimulation* * *task* interaction was significant [*F*(1,21) = 7.02, *p* = 0.015, 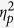 = 0.2505]. A paired-samples t-test with Bonferroni correction revealed that rhythm memory performance was facilitated when receiving anodal tDCS over the right SMG [*t*(21) = 2.78, *p* = 0.011, 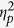 = 0.269] (Fig. 1). No significant modulation effect was found for pitch memory [*t*(21) = 1.68, *p* = 0.108, 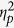 = 0.118].

In addition, separate ANOVAs for each task were conducted with stimulation and group as the independent variables. For the pitch memory task the analysis showed non-significant main effects of *stimulation* [*F*(1,40) = 0.39, *p* = 0.538, 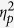 = 0.010] and *group* [*F*(1,40) = 1.95, *p* = 0.170, 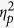 = 0.047] and a significant *stimulation* * *group* interaction [*F*(1,40) = 12.5, *p* = 0.004, 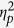 = 0.185]. The ANOVA for the rhythm task revealed a trend for the factor *stimulation* [*F*(1,40) = 2.97, *p* = 0.093, 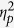 = 0.069], a non-significant effect of *group* [*F*(1,40) = 0.57, *p* = 0.453, 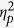 = 0.014] and a non-significant *stimulation* * *group* interaction [*F*(1,40) = 2.60, *p* = 0.115, 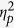 = 0.061].

## Discussion

The aim of the study was to investigate the involvement of the left and right SMG for pitch and rhythm memory, and to explore whether a hemispheric distinction can be found depending on the task. Experiment 1 revealed that anodal tDCS over the left SMG significantly facilitated pitch memory, whereas rhythm memory was not affected. Experiment 2 showed that anodal tDCS over the right SMG facilitated rhythm memory performance, whereas pitch memory performance was not modulated. The study highlights a different hemispheric involvement of the SMG for rhythm and pitch memory.

The finding that the left but not right SMG is causally involved in pitch memory is as hypothesised and in accordance with previous studies^38^,^40^,^41^. Since rhythm memory was not modulated by tDCS over the left SMG, the results of Experiment 1 also reveal that the left SMG is not causally involved throughout the auditory memory domain. The selective improvement of pitch but not rhythm memory suggests that the function of the left SMG is restricted to pitch information in auditory memory. As the left SMG has also been shown to be involved in verbal memory tasks^44^,^45^, one might also conclude that the left SMG relates to general pitch-based memory functions such as tonal pitch or intonation memory. This will be an interesting avenue to explore in future studies. For instance, does tDCS to the left SMG specifically modulate pitch memory span, versus verbal span. In previous studies, we have shown that tDCS over the left and right SMG did not modulate memory performance on a visual memory task^38^,^40^, but future work should address the role of the left SMG in verbal memory span performance.

When anodal tDCS was applied over the right SMG in Experiment 2, a different pattern was revealed. No modulation was shown for pitch memory which is in accordance with a previous study of our group^40^. Interestingly, improved performance on the rhythm memory task was found, indicating that the right SMG is related to rhythm memory. This is in accordance with fMRI studies showing a dominant rightward activation of the SMG for rhythm discrimination and memory^12^,^42^. To the best of our knowledge, this is the first study revealing a causal relationship of a particular brain area (i.e. the right SMG) for rhythm memory by means of non-invasive brain stimulation.

The study shows a dissociation of the involvement of the left and right SMG for pitch and rhythm memory. Hemispheric differences of the involvement of the SMG have also been shown depending on expertise. In a previous study we showed that cathodal tDCS over the left SMG led to a deterioration of pitch memory performance in non-musicians, whereas musicians showed a decline in pitch memory after cathodal tDCS over the right SMG^40^. This highlights a hemispheric shift of the involvement of the SMG with musical training, which may be due to different strategies used for memorising the pitch information between non-musicians and musicians. Furthermore, Herdener *et al*.^46^ revealed that rhythm processing activates a network predominantly in the right hemisphere, but that highly trained drummers additionally show activation in the left SMG. These authors highlight a link between the left SMG and linguistic syntax processing, which would suggest that drummers try to give a meaning to the rhythmical cues. It would be interesting to perform the present study protocol with musicians as participants in order to further investigate the significance of the left and right SMG for pitch and rhythm memory taking into account musical expertise.

As it is expected that the tDCS effects are the strongest under the active electrode, the results of our study highlight the significance of the left SMG for pitch memory as well as linking the right SMG to rhythm memory. It is, however, possible that the effects also spread to brain areas that are functionally connected with the stimulated area^47^, and that this also influences the behavioural performance. As the literature highlights a strong connection of the right inferior and frontal areas for rhythm memory^12^,^13^, it might be that the memory enhancement may also be due to an increased interconnection of the right SMG to frontal areas. For pitch memory the spread of activation could have strengthened the connections of the left SMG to frontal or auditory cortices^11^. Assessing the impact of brain stimulation on functional interactions within and between brain networks is an important next step for future research^27^.

In sum the study highlights a hemispheric dissociation of the SMG for different auditory materials in non-musicians. Whereas the significance of the left SMG can be linked to pitch memory performance, the right SMG seems to be involved in rhythm memory.

## Methods

### Participants

Forty-four right-handed non-musicians (10 male) were recruited for this study. Two participants were excluded for the analysis. One participant indicated that she used the keys the wrong way round in the rhythm span task and one participant was excluded as her rhythm memory performance during sham stimulation was identified as an extreme outlier using the Grubbs Test^48^. The remaining sample for the analysis consisted of 20 participants (5 male) in Experiment 1 and 22 participants (5 male) in Experiment 2. Participants did not play an instrument currently and had less than two years of musical training in the past. The minimal musical training exposure was confirmed by a low mean score of 14.17 points (SD = 5.04) in the Musical Training Dimension (possible range 7–49, see Material for more information) of the German version of the Goldsmiths Musical Sophistication Index (Gold-MSI)^49^,^50^. The samples of both experiments were matched by age, gender and Gold-MSI score (see Table 1 for demographical details).

The study was approved by the ethics committee of the Medical Department of the Heinrich-Heine-University in Düsseldorf and the methods were carried out in accordance with the Declaration of Helsinki. All participants gave their informed written consent to take part prior to the study.

## Material

### Pitch and rhythm memory task

In order to measure pitch memory abilities, the pitch span task^51^ was used. This task measures pitch memory capacity by identifying the maximum span of tones the individual participant can keep in mind. The task uses 10 triangle-waveform tones (equally tempered, whole tone steps) with fundamental pitches ranging from 262 Hz (C4) to 741 Hz (F#5) which are 500 msec long and which are presented with 383 msec pause between each other. To begin with participants hear two pitch sequences which are two tones long with a 2 sec pause between sequences and the task is to indicate whether the two sequences are the same or different. The sequence length increases and decreases using an adaptive staircase procedure. When participants give two correct answers, one tone is added and when participants give one wrong answer, one tone is taken away. By using this procedure, participants are pushed to their limit of pitch memory capacity. The task is completed when 6 reversals are reached^51^.

For the evaluation of rhythm memory, the rhythm span task^52^ was used. This task was developed following the experimental parameters of the described pitch memory task. Instead of presenting the participants with tone sequences, two rhythm sequences were played and the task was to judge whether the rhythms were the same or different. Six rhythm elements were created. Elements were 1 second long (spanning over one quarter note) and contained one to three units (quarter notes, eighth notes, sixteenth notes and eighth note triplets), all presented on the same pitch. As the precise timing is important, 20 sequences were created (10 same and 10 different) for each sequence lengths (2 to 10 elements). Rhythm elements were randomly sampled and the span task followed the same adaptive staircase procedure as the pitch memory task. Participants were instructed to press the left command button if they thought the sequences were the same and the right command button for different sequences^52^.

### Gold-MSI questionnaire

The German version of the self-report questionnaire of the Goldsmiths Musical Sophistication Index v1.0 (Gold-MSI)^49^,^50^ was used to evaluate musical training and sophistication in order to ensure that only non-musicians took part. The questionnaire evaluates musical behaviour and engagement and participants are asked to rate 38 statements using a seven-point scale. The Gold-MSI comprises a general factor ‘Musical Sophistication’ as well as five individual dimensions: Active Engagement, Perceptual Abilities, Musical Training, Emotions and Singing Abilities. The dimension of interest for this study is Musical Training which includes seven items and a possible score of 7–49 points.

### TDCS parameters

TDCS was applied over the left SMG in Experiment 1 and the right SMG in Experiment 2. In Experiment 1, the left SMG was identified using CP3 of the international 10–20 system for electroencephalogram electrode placement. In Experiment 2, the right SMG was located using CP4. This method has been used successfully in previous studies^40^,^41^. An active electrode (5 cm × 5 cm = 25 cm^2^) was placed over the targeted site, left or right SMG respectively, and the reference electrode (5 cm × 7 cm = 35 cm^2^) was adjusted over the contralateral supraorbital area. The electrodes were covered in saline-soaked sponges and fixed on the scalp using self-adhesive bandages. For the active condition 15 minutes of anodal tDCS (with 30 seconds fade-in and fade-out) with an intensity of 2 mA was applied. For the sham condition, an identical set-up was used but the stimulation only lasted for 30 seconds (with additional 30 seconds fade-in and fade-out). This evokes the sensation of being stimulated but does not lead to any neurophysiological changes^53^.

### Procedure

The study comprises two experiments which were identical in their procedure except that a different target brain area, left or right SMG, was stimulated. In Experiment 1, anodal or sham tDCS was applied over the *left* SMG and in Experiment 2 stimulation was applied over the *right* SMG.

Each participant took part in two sessions and either received anodal or sham stimulation over the targeted area. The order of stimulation was counterbalanced between participants. After signing the consent form the electrodes were placed on the scalp and the stimulation began. During the 15 minutes of stimulation participants were asked to sit back and relax. As soon as the stimulation finished, the participants completed the pitch and rhythm span task. The order of tasks was counterbalanced between participants. At the end of the first session the participants filled in the Gold-MSI questionnaire.

## Additional Information

**How to cite this article:** Schaal, N. K. *et al*. Hemispheric differences between left and right supramarginal gyrus for pitch and rhythm memory. *Sci. Rep.* **7**, 42456; doi: 10.1038/srep42456 (2017).

**Publisher's note:** Springer Nature remains neutral with regard to jurisdictional claims in published maps and institutional affiliations.

## Figures and Tables

**Table 1:** Demographical details for the sample of the two experiments.

	N	Age	Gender (m/f)	Gold-MSI Musical Training
Experiment 1	20	22.80 (±4.16)	5 m/15 f	14.55 (±5.04)
Experiment 2	22	22.59 (±3.08)	5 m/17 f	13.82 (±5.12)
